# Alu and B1 Repeats Have Been Selectively Retained in the Upstream and Intronic Regions of Genes of Specific Functional Classes

**DOI:** 10.1371/journal.pcbi.1000610

**Published:** 2009-12-18

**Authors:** Aristotelis Tsirigos, Isidore Rigoutsos

**Affiliations:** Bioinformatics and Pattern Discovery Group, IBM Thomas J. Watson Research Center, Yorktown Heights, New York, United States of America; Washington University School of Medicine, United States of America

## Abstract

Alu and B1 repeats are mobile elements that originated in an initial duplication of the 7SL RNA gene prior to the primate-rodent split about 80 million years ago and currently account for a substantial fraction of the human and mouse genome, respectively. Following the primate-rodent split, Alu and B1 elements spread independently in each of the two genomes in a seemingly random manner, and, according to the prevailing hypothesis, negative selection shaped their final distribution in each genome by forcing the selective loss of certain Alu and B1 copies. In this paper, contrary to the prevailing hypothesis, we present evidence that Alu and B1 elements have been selectively retained in the upstream and intronic regions of genes belonging to specific functional classes. At the same time, we found no evidence for selective loss of these elements in any functional class. A subset of the functional links we discovered corresponds to functions where Alu involvement has actually been experimentally validated, whereas the majority of the functional links we report are novel. Finally, the unexpected finding that Alu and B1 elements show similar biases in their distribution across functional classes, despite having spread independently in their respective genomes, further supports our claim that the extant instances of Alu and B1 elements are the result of positive selection.

## Author Summary

Despite their fundamental role in cell regulation, genes account for less than 1% of the human genome. Recent studies have shown that non-genic regions of our DNA may also play an important functional role in human cells. In this paper, we study Alu and B elements, a specific class of such non-genic elements that account for ∼10% of the human genome and ∼7% of the mouse genome respectively. We show that, contrary to the prevailing hypothesis, Alu and B elements have been preferentially retained in the proximity of genes that perform specific functions in the cell. In contrast, we found no evidence for selective loss of these elements in any functional class. Several of the functional classes that we have linked to Alu and B elements are central to the proper working of the cell, and their disruption has previously been shown to lead to the onset of disease. Interestingly, the DNA sequences of Alu and B elements differ substantially between human and mouse, thus hinting at the existence of a potentially large number of non-conserved regulatory elements.

## Introduction

Identifiable repeat elements cover a very large fraction of the human and mouse genomes, and even though they are quite diverse at the sequence level, they can be assigned to a fairly small number of families [1]. Alu and B elements belong to the Short Interspersed Nuclear Element (SINE) family, members of which exist in several mammalian genomes, where they have spread in great copy numbers [2]–[4]. Alu elements, the most abundant class or repeat elements in the human genome, originated in the duplication and subsequent fusion of the 7SL RNA gene at the beginning of the radiation of primates [5],[6]. B1 elements belong to the same repeat family and have also descended from the 7SL RNA. Following the primate-rodent split, copies of Alu and B1 elements have amplified and duplicated *independently* in the two genomes while accumulating mutations [4],[7]. The extent of the acquired mutations is such that extant instances of archetypal Alu and B1 elements bear little resemblance to one another or to the original 7SL RNA gene.

In earlier work, the Alu distribution in the human genome was studied in terms of several genomic features in order to understand how they spread in the genome: it was shown that Alu elements are predominant in R bands and inversely distributed with respect to L1 elements [8], correlated with GC-rich parts of the genome [9],[10] as well as gene and intron density [10]–[12], and enriched in isochores [11], segmental duplications [13] and transcription factor binding sites [14]. Another study of Alu, B1 and related SINE elements across mammalian genomes demonstrated their presence in primates, rodents, and tree-shrews and their absence in other mammals [15]. There have also been attempts to associate Alu elements with functional classes of genes. In [16], Alu elements located on chromosomes 21 and 22, were found to be over-represented in a limited set of functional classes. Housekeeping genes vs. tissue-specific genes were also found to have preferences for Alu elements [17]. In [14], the authors considered for their analysis only 5 kb upstream of known genes, and a limited set of functional classes for over-representation or under-representation of Alu elements.

In what follows, we extend previous work by studying and comparing the distributions of extant instances of both Alu and B1 elements, as well as related B2 and B4 elements (from this point on, we will be referring to B1, B2 and B4 elements collectively as “B elements”) in *both upstream and intronic regions* of known protein-coding genes, in order to contribute to the understanding of the *evolutionary history* of these elements. More specifically, we test whether their current distributions in the human and mouse genomes are a result of positive or negative selection across functional classes of genes.

## Results

### Alu and B element densities vary as a function of their upstream/downstream distance from gene transcript start positions

Following the primate-rodent split, Alu and B elements spread throughout the human and mouse genomes: Alu elements currently number ∼1.1 million copies and cover about 5.4% of the human genome (in the sense orientation), while B elements number ∼1.2 million copies and cover about 3.6% of the mouse genome (in the sense orientation).

We studied Alu and B element densities separately for all combinations of: (a) *distance* from gene transcript start positions, (b) *direction* (upstream vs. downstream), and (c) *orientation* (sense vs. antisense). In the case of downstream direction, we computed Alu and B element densities separately for intronic and exonic regions. For a detailed description of the computation method and all relevant definitions, see Methods section. Our results demonstrate that Alu and B elements are significantly over-represented in the *upstream* regions of genes, and that the highest densities are observed within the window ending at 16 kb upstream of gene transcript start positions. For a detailed explanation of how we determine *significance* and how we compute *p-values* for all cases of over-representation and under-representation, see Methods. Similarly, Alu and B elements are significantly over-represented in the *intronic downstream* regions of genes, and, just as in the upstream case, the highest densities are observed in the window ending at 16 kb downstream of the gene transcript start positions. However, in introns, the over-representation is significantly more pronounced in the antisense orientation. Finally, there is a significant under-representation of Alu and B elements in *exons* and the effect of distance is not as pronounced as in the upstream and intronic downstream cases.

**Figure 1:**
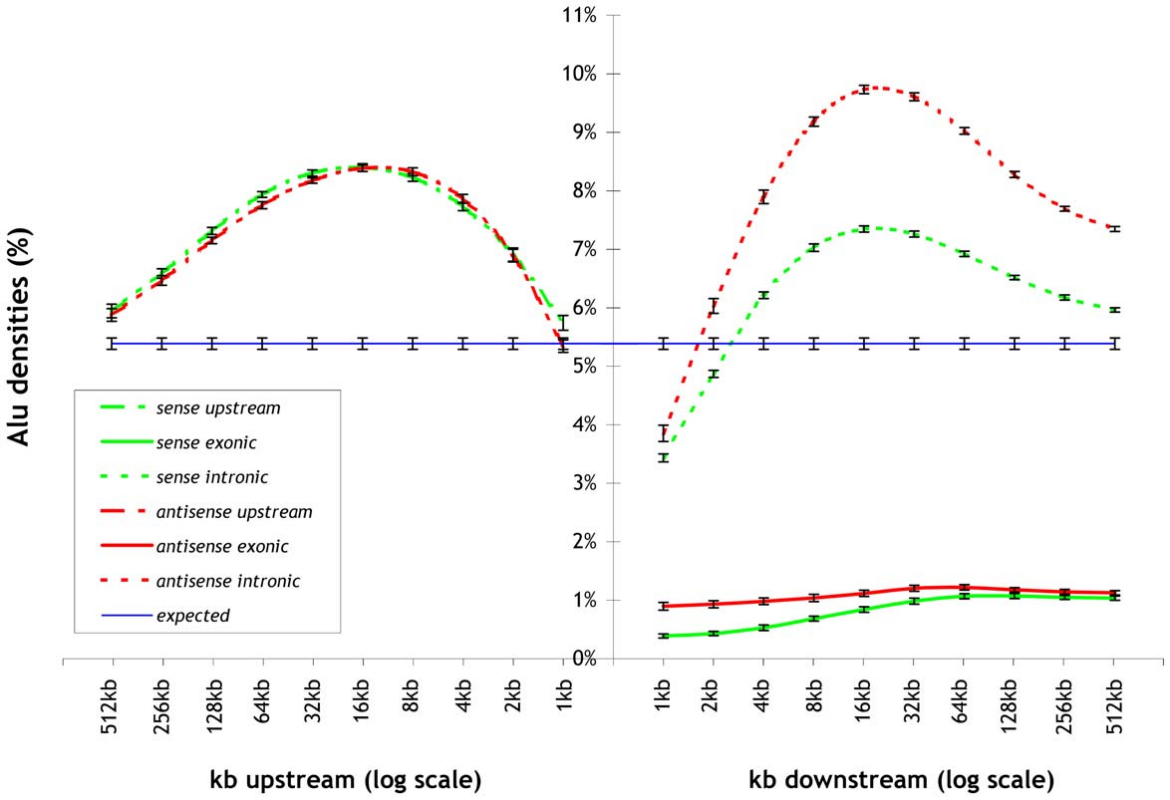
Alu densities upstream and downstream of known genes as a function of distance from the gene transcript start position. Green and red curves correspond to Alu instances in the sense and antisense orientation respectively. Downstream regions are separated in exonic and intronic parts. There is a clear over-representation of Alu instances upstream of known genes and in the intronic regions, particularly in the antisense direction. In contrast, Alu elements are under-represented in exons.

**Figure 2:**
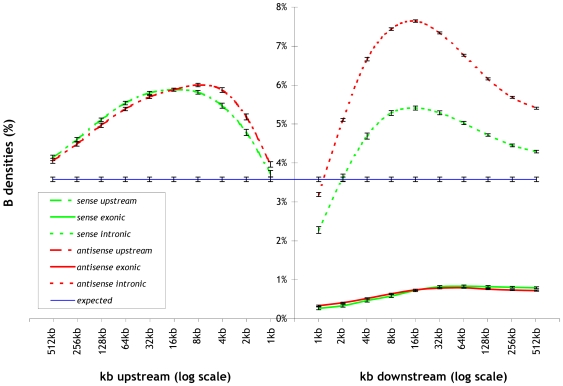
B element (B1, B2, B4) densities upstream and downstream of known genes as a function of distance from the gene transcript start position. Green and red curves correspond to B element instances in the sense and antisense orientation respectively. Downstream regions are separated in exonic and intronic parts. As in the case of Alu elements, there is a clear over-representation of B element instances upstream of known genes and in the intronic regions, particularly in the antisense direction. In contrast, B elements are under-represented in exons.

These results are shown in detail in Figure 1 for Alu elements in human and in Figure 2 for B elements in mouse: we plot Alu and B element densities upstream and downstream of known genes as a function of distance from the gene transcript start positions. Green and red curves correspond to Alu and B densities in the sense and antisense orientation respectively. In the downstream case, we distinguish between exonic and intronic regions.

### Human Alu elements have been selectively retained in upstream and intronic regions of genes of specific functional classes

We first associated Alu elements to functional classes by performing a genome-wide analysis on the latest release of the human genome annotations and applying a distribution-free statistical test with multiple hypothesis testing correction. Unlike the analysis in [14], where only 5 kb upstream of known genes were considered, we examined the 0 kb–16 kb window for the upstream analysis, i.e. the window where we find that the Alu density is maximized (see above). In addition, we: (a) examined the possibility that intronic instances might also be linked to specific functional classes, and (b) treated sense and antisense orientations separately. As a result, we were able to associate with Alu elements at least four times more functional classes than we would have been able to, had we only considered 5 kb upstream regions. Finally, after determining the functional associations, we conducted *additional* computational experiments to pinpoint the most likely explanation for the observed functional biases.

We applied the following statistical test in order to determine potential biases in the positioning of Alu elements within upstream and intronic regions of genes belonging to specific functional classes. After labeling each gene's upstream or intronic region with the GO terms attributed to the corresponding spliced transcripts, we tested whether Alu densities are *significantly higher* in the upstream or intronic regions of genes associated with certain GO terms. *Density* is defined as the fraction of the upstream or intronic region of a given gene that is covered by Alu instances. For a more formal definition of density and a detailed description of the statistical method used here we refer the reader to the Methods section.

Using this approach we found that upstream and intronic Alu instances are not randomly distributed, but instead are located, significantly more frequently than expected, inside upstream and intronic regions (in either the sense or antisense direction) of genes belonging to specific functional classes, i.e. GO terms. In Table 1, we report these functional classes at GO hierarchy level six or greater. In Supplemental Table S1, we report the entire list of GO terms and the associated p-values.

**Table 1:** Significantly over-represented GO terms for Alu and B elements.

	Human Alu				Mouse B				
GO term id	genes	U	I+	I-	genes	U	I+	I-	GO term description
GO:0016279	29	√			24	√		√	protein-lysine N-methyltransferase activity
GO:0018024	29	√			24	√		√	histone-lysine N-methyltransferase activity
GO:0042054	37	√			33	√		√	histone methyltransferase activity
GO:0016278	29	√			24	√		√	lysine N-methyltransferase activity
GO:0004713	556	√			572	√			protein-tyrosine kinase activity
GO:0004674	541	√			564	√		√	protein serine/threonine kinase activity
GO:0017111	761	√			725	√	√	√	nucleoside-triphosphatase activity
GO:0016887	378	√			363	√	√	√	ATPase activity
GO:0042623	292	√			274	√	√	√	ATPase activity, coupled
GO:0003924	261	√			241	√			GTPase activity
GO:0004721	174	√			161	√			phosphoprotein phosphatase activity
GO:0004842	161	√			151	√	√	√	ubiquitin-protein ligase activity
GO:0030983	23	√	√	√	12				mismatched DNA binding
GO:0045934	389	√			357	√			negative regulation of nucleobase, nucleoside, nucleotide and nucleic acid metabolism
GO:0051053	23	√			18				negative regulation of DNA metabolism
GO:0008156	18	√			12				negative regulation of DNA replication
GO:0016481	358	√			335	√			negative regulation of transcription
GO:0045449	2723	√		√	2515	√		√	Regulation of transcription
GO:0006355	2554	√		√	2363	√		√	Regulation of transcription, DNA-dependent
GO:0051052	73	√			48	√			Regulation of DNA metabolism
GO:0006445	60	√			31	√			Regulation of translation
GO:0006446	44	√			22	√			Regulation of translational initiation
GO:0043065	299	√			254	√			positive regulation of apoptosis
GO:0006917	250	√			190	√			induction of apoptosis
GO:0012502	251	√			190	√			induction of programmed cell death
GO:0043066	276	√			226				negative regulation of apoptosis
GO:0043414	50	√			74				biopolymer methylation
GO:0043037	263	√		√	165	√	√	√	translation
GO:0006414	108	√			24				translational elongation
GO:0006413	69	√			65	√			translational initiation
GO:0043632	237	√		√	162	√	√	√	modification-dependent macromolecule catabolism
GO:0019941	237	√		√	162	√	√	√	modification-dependent protein catabolism
GO:0006511	234	√		√	159	√	√	√	ubiquitin-dependent protein catabolism
GO:0043161	100	√			29				proteasomal ubiquitin-dependent protein catabolism
GO:0030433	18	√			11	√			ER-associated protein catabolism
GO:0006401	51	√			35				RNA catabolism
GO:0006402	34	√			29				mRNA catabolism
GO:0000184	21	√		√	16	√			mRNA catabolism, nonsense-mediated decay
GO:0044257	262	√		√	185	√	√	√	cellular protein catabolism
GO:0051603	259	√		√	183	√	√	√	proteolysis during cellular protein catabolism
GO:0006515	18	√			12	√			Misfolded or incompletely synthesized protein catabolism
GO:0016310	878	√			873	√			phosphorylation
GO:0006468	727	√			750	√			protein amino acid phosphorylation
GO:0006310	112			√	86	√	√	√	DNA recombination
GO:0006260	223	√	√	√	167	√	√	√	DNA replication
GO:0006261	120	√	√	√	74	√			DNA-dependent DNA replication
GO:0045005	31		√	√	15				maintenance of fidelity during DNA-dependent DNA replication
GO:0006323	418	√		√	378	√	√	√	DNA packaging
GO:0006325	414	√		√	376	√	√	√	establishment and/or maintenance of chromatin architecture
GO:0016568	216	√		√	192	√	√	√	Chromatin modification
GO:0016569	58	√			55	√			covalent chromatin modification
GO:0006338	56	√		√	49	√		√	Chromatin remodeling
GO:0006396	504	√	√	√	411	√	√	√	RNA processing
GO:0006397	307	√	√	√	244	√	√	√	mRNA processing
GO:0000398	161	√	√	√	51				nuclear mRNA splicing, via spliceosome
GO:0000387	28	√	√		4				spliceosomal snRNP biogenesis
GO:0000245	36	√			20				spliceosome assembly
GO:0008380	278	√	√	√	194	√		√	RNA splicing
GO:0000375	161	√	√	√	51				RNA splicing, via transesterification reactions
GO:0000377	161	√	√	√	51				RNA splicing, via transesterification reactions with bulged adenosine as nucleophile
GO:0043631	12	√			13				RNA polyadenylation
GO:0016071	352	√	√	√	286	√	√	√	mRNA metabolism
GO:0006351	2629	√		√	2408	√		√	transcription, DNA-dependent
GO:0006352	111	√		√	64			√	transcription initiation
GO:0006367	70	√		√	23				transcription initiation from RNA polymerase II promoter
GO:0006354	52	√		√	11				RNA elongation
GO:0006368	49	√		√	7				RNA elongation from RNA polymerase II promoter
GO:0006366	736	√			579	√			transcription from RNA polymerase II promoter
GO:0006508	868	√			827				proteolysis
GO:0006457	203	√			150	√		√	protein folding
GO:0006464	1918	√		√	1805	√	√	√	protein modification
GO:0043543	32	√			27				protein amino acid acylation
GO:0006473	23	√			16				protein amino acid acetylation
GO:0006512	603	√		√	552	√		√	ubiquitin cycle
GO:0031365	11	√			7				N-terminal protein amino acid modification
GO:0008632	108	√			78	√			Apoptotic program
GO:0051170	107	√			83	√			nuclear import
GO:0006606	105	√			81	√			protein import into nucleus
GO:0051168	55	√			41	√	√		nuclear export
GO:0006405	36	√			21				RNA export from nucleus
GO:0006605	222	√			228	√		√	protein targeting
GO:0051028	80	√		√	55	√	√	√	mRNA transport
GO:0007067	224	√		√	192	√	√	√	Mitosis
GO:0051437	72	√			0				positive regulation of ubiquitin ligase activity during mitotic cell cycle
GO:0007017	231	√			219	√	√	√	microtubule-based process
GO:0007001	442	√		√	402	√	√	√	chromosome organization and biogenesis (sensu Eukaryota)
GO:0030520	10	√			4				estrogen receptor signaling pathway

In the interest of clarity of the presentation we *only* show GO terms at GO hierarchy level ≥6; the entire list of GO terms can be found in Supplemental Table S1. The colors in the columns labeled “Alu” and “B” show for each GO term whether it is associated with upstream (U), sense intronic (I+), or antisense intronic (I-) regions. Significant GO terms are considered those terms whose *adjusted p-values* are less than 0.01 (see Methods). The actual adjusted and unadjusted p-values for each type of element and for each region and orientation can be found in Supplemental Table S1. The *GO terms are organized* in such a way so that related GO terms are located as close as possible to one another (note that this is not an easy problem, since the GO hierarchy is not a tree).

**Figure 3:**
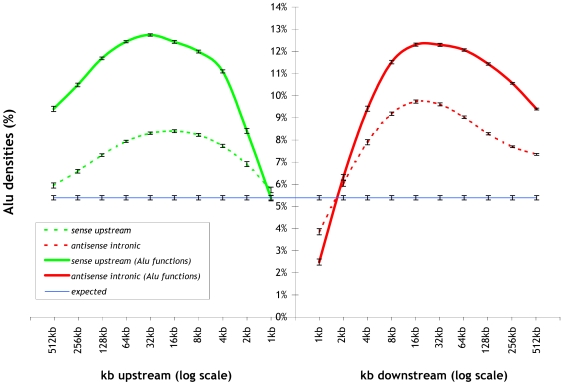
Alu densities upstream and downstream of known genes as a function of distance from the gene transcript start position. Green and red curves correspond to Alu instances in the sense and antisense orientation respectively. Here, we show only the subset of the curves of Figure 1 with the highest densities, i.e. sense upstream and antisense intronic downstream, and compare it the corresponding densities of the genes which belong to the experimentally validated functional classes: DNA repair, DNA recombination, chromatin remodeling, splicing and translation.

In order to validate our computational findings, we searched the existing literature for *experimental evidence* linking Alu elements to specific functions and compared them to the GO terms listed in Table 1 (or in the full list of significant GO terms found in Supplemental Table S1). Alu elements have been shown to be involved in DNA repair [18], to play a role in alternative splicing, RNA editing and translation regulation [19],[20], to repress transcription following heat shock [21], and to affect genomic organization and evolution, through insertion mutation and recombination [4],[22]. For most of these functions, we were able to find related significant GO terms: DNA repair, RNA splicing, translation, chromatin remodeling, and DNA recombination. In Figure 3, we verify that for these GO terms, the Alu density of associated genes in upstream and intronic regions is significantly higher than we would expect in a randomly chosen set of genes. Interestingly, most of the functional classes reported in Table 1 have not previously been linked to Alu elements, suggesting potential novel regulatory roles for these elements.

In search for the most likely interpretation of the functional biases of Alu instances in upstream and intronic regions reported in Table 1, we explored three alternative scenarios, and conducted further computational experiments in order to prove or disprove them. One possible explanation for our findings could be that Alu elements were *selectively retained* through natural selection in the genes of these functional classes, because they play a positive role in the function of these genes and offer a selective advantage. Had these insertions been neutral, no functional biases would have been observed in our analysis. If, on the other hand, these insertions had had a negative impact, they would have been selected against during evolution, considering that insertions in upstream regions of genes, where regulatory signals are located, could easily disrupt normal function. Not surprisingly, an obvious case of negative selection is found in the *exonic* regions where not only Alu elements are under-represented (see Figure 1), but also no functional biases are observed, in other words, the negative selection of Alu elements in exonic regions is active across all functional classes.

A second possible explanation could be that mobile elements in general possess either an insertion or a tolerance bias towards these functional classes of genes. In other words, either mobile elements may be preferentially inserted in genes belonging to these functional classes, or genes in these functional classes may tend to tolerate mobile element insertions better than the rest of the genes. To corroborate or refute these hypotheses, we tested whether other types of mobile repeat elements are enriched in the same functional classes as Alu elements and, in general, we found *no significant overlap*: 22% with LINEs and 1% with ERVs, 1% with LTRs and zero for all other mobile element families. Even in the case of LINEs, where we observed the highest overlap, none of these common classes is related to DNA repair, recombination, chromatin remodeling, splicing or translation. In addition, we analyzed the three main Alu subfamilies and discovered *significantly fewer* functional biases for the *recently inserted* Alu elements (see following section), thus demonstrating that these functional biases are crystallized as Alu elements survive longer inside the genome, and after some of these elements have been retained. In summary, we conclude that Alu elements share little in common in terms of functional biases with either older or younger mobile elements, and we can therefore rule out the tolerance and preferential insertion hypotheses, a conclusion that is in fact consistent with previous findings [23],[24].

A third alternative explanation could be that certain Alu instances were selectively lost after the initial random spreading, and, in fact, this scenario corresponds to the prevailing hypothesis. However, when we tested whether Alu elements are under-represented in the upstream or intronic regions of genes of specific functional classes, we *found no such bias*. This suggests that Alu instances have been lost *randomly* across functional classes.

Based on the above analysis, we conclude that, as described in the first scenario, there has been a positive selection of Alu elements in the upstream and intronic regions of the genes that belong to the functional classes reported in Table 1. This finding suggests that Alu elements likely play an active role in the entire set of functions listed in Table 1, and not only in the small subset which has already been reported in the literature.

### Mouse B1 elements have *independently* been retained in the upstream and intronic regions of genes of similar functional classes to human Alu elements

**Figure 4:**
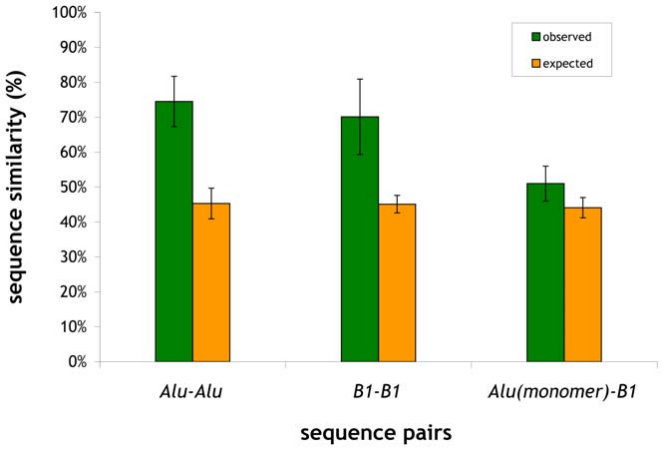
Average pair-wise sequence similarities involving Alu and B1 elements. We have carried out pair-wise comparisons involving a) only Alu elements, b) only B1 elements, and c) Alu monomers with B1 elements.

B1 and Alu repeat families both descended from an initial duplication of the 7SL RNA gene [4] before the primate-rodent split, i.e. more that 80 million years ago. However, after the primate-rodent split, Alu and B elements spread *independently*, accumulated mutations and, over time, substantially diverged from the 7SL RNA sequence from which they originated [4],[7]. Consequently, extant B1 elements should be very different from Alu elements at the sequence level. We confirmed the lack of sequence similarity between Alu and B1 elements in two ways. First, in Figure 4, we show that the average pair-wise similarity among Alu elements is 71.5±11.1%, whereas the expected similarity is 45.3±4.4% as determined using shuffled versions of the Alu sequences. The average pair-wise similarity for B1 elements is 70.1±10.8%, whereas the expected similarity is 45.1±2.5%. In contrast, the average pair-wise similarity between extant Alu monomers and B1 elements is only 51.1±4.7% and very close to the expected similarity value of 44.2±2.7%. Second, using human/mouse whole-genome alignments we found that Alu and B1 elements are located overwhelmingly in non-conserved regions of the human and mouse genomes: the percentages are ∼99.9% in the case of Alu elements and ∼96.4% in the case of B elements (∼95.8% for B1, ∼96.9% for B2 and ∼96.5% for B4 elements).

Next, we applied the same statistical analysis used in the previous section, in order to look for enrichment of B elements in specific functional classes of genes. Given that, as shown above, the sequences of B elements are so *different* from those of Alu elements, and that the current distribution of Alu and B elements has been shaped *independently* in the each of the two genomes through initial random spreading and subsequent loss of certain copies, one would expect that the functional associations of B elements in upstream and intronic regions of genes would be different from the ones described in the previous section. However, we found that the set of functions associated with B elements contains 83.2% of the functions associated with Alu elements (expected = 12.2±2.0%). The fact that this result is observed independently in the mouse genome further strengthens our claim that these two types of SINE elements have been *selectively retained* in genes of certain functional classes, rather than selectively lost from certain genes.

Nevertheless, we examined an alternative scenario: since Alu and B elements are found in non-conserved regions of human and mouse, we tested whether certain functional classes of genes tend to have non-conserved upstream and intronic regions (effectively defining the differences between these two organisms), and whether these functional classes overlap with those associated with Alu and B elements. We found that the set of GO terms associated with non-conserved regions and the set of GO terms associated with Alu elements share *only five* entries in the combined sense/antisense intronic regions, and *zero* in the combined sense/antisense upstream regions. The common GO terms in the intronic case are generic high-level terms (e.g. metabolism, binding, etc.), and do not include DNA repair, recombination, chromatin remodeling, splicing or translation. Therefore, we conclude that lack of conservation of Alu and B elements does not explain the observed functional biases.

### Functional biases of Alu and B element instances extend to all Alu and B element sub-families

**Figure 5:**
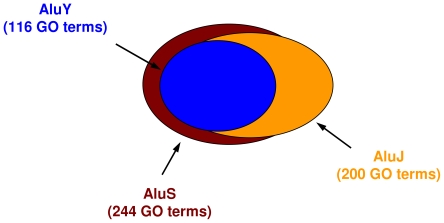
Venn diagram showing the relationships among the three sets of significant GO terms corresponding to each Alu sub-family. Note that the AluS GO term set is an approximate superset of the AluJ set, which in turn is an approximate superset of the AluY set – see test for details.

Human Alu elements belong to one of three main sub-families AluS, AluJ and AluY, with approximately 660,000, 283,000 and 148,000 copies respectively in the human genome. We repeated the above GO term analysis separately for each Alu sub-family and found that all three Alu sub-families are significantly over-represented in the upstream and intronic regions of genes of certain functional classes. Using the same cutoff on the adjusted p-values, we obtained 244 significant GO terms for the oldest AluS sub-family, 200 for the AluJ sub-family and 116 for the youngest AluY sub-family. The relationships of these three sets to one another are depicted in the form of a Venn diagram in Figure 5. A qualitative interpretation of the Venn diagram is that the AluS GO term set is an approximate superset of the AluJ set (86.0% of the AluJ set members are also members of the AluS set; expected overlap is 7.7±1.6%), which in turn is an approximate superset of the AluY set (93.1% of the AluY set members are also members of the AluJ set; expected overlap is 6.6±2.3%). The AluY set is 100% covered by the AluS set. The computed p-values for all sub-families, for both upstream and intronic regions, and for both sense and antisense orientations can be found in the Supplemental Tables S2 and S3.

Similarly, in the mouse genome there are B1, B2 and B4 elements with approximately 417,000, 363,000 and 390,000 copies respectively. Using the same method and cutoff, we found 293, 260 and 232 significant GO terms for B1, B2 and B4 elements respectively. Unlike Alu sub-families, where the number of significant GO terms increased with the age of the sub-family, here all three types of elements have comparable numbers of significant GO terms associated with them. Also, pair-wise intersection of these lists of GO terms show high similarities, measured using the Jaccard coefficient between each pair of sets: 65.6% between B1 and B2 (expected similarity = 4.6±0.8%), 54.0% between B1 and B4 (expected similarity = 4.4±0.9%), and 56.2% between B2 and B4% (expected similarity = 4.0±0.9%). The computed p-values for B1, B2 and B4 elements, for both upstream and intronic regions, and for both sense and antisense orientations can be found in the Supplemental Tables S4 and S5.

### Alu and B elements in other organisms

Almost all instances of Alu elements in human (95%) are conserved in the chimpanzee genome, i.e. they are included in human-chimpanzee whole-genome alignments. After repeating the GO analysis in the chimpanzee genome, we concluded that 81% of the identified significant GO terms are identical to the significant GO terms identified in human. Similarly, B elements are conserved between mouse and rat genomes: 50% of B element instances in mouse have a conserved counterpart in rat. Even though the level of conservation between mouse and rat B elements is not as high as between human and chimpanzee Alu elements, 90% of the significant GO terms identified in rat genome are identical to the significant GO terms identified in mouse. The results of the chimpanzee and rat analyses can be found in the Supplemental Tables S6 and S7 for chimpanzee and Tables S8 and S9 for rat. In conclusion, our findings show that there exists a human-chimpanzee conservation of Alu elements and a mouse-rat conservation of B elements on the sequence level. More importantly, there exists a conserved *functional* connection between all four organisms, independent of the level of cross-species sequence conservation of these elements.

## Discussion

Our analyses reveal that *both upstream and intronic regions* in human and mouse are significantly enriched in Alu and B elements respectively. Surprisingly, we find that Alu and B elements are significantly enriched across similar functional classes in human and mouse, even though these two types of elements have spread independently in the two genomes, following the primate-rodent split. In contrast, we find no depletion across functional classes, a finding which suggests that the final distribution of Alu and B elements across the two genomes is unlikely to be the result of a selective loss of some of their randomly retrotransposed copies. A simpler explanation suggests that they have been *selectively retained* in the upstream and intronic regions of genes belonging to the functional classes presented in Table 1, presumably because they offered some selective advantage (for example more binding sites to help increase the complexity of regulatory networks, or more transcript splice variants) thus increasing each organism's chances of survival. Indeed, a subset of the functional associations we uncovered in this paper has been reported in the literature, thus supporting the merit of our computational approach, while the majority of the functions are novel and suggest possible avenues to specific experimental tests.

Most importantly, our analysis suggests that SINEs are *implicated in gene regulation* effected through the upstream and intronic regions of specific genes, and contributes to an increasing body of literature attributing functional relevance to repeat elements which were initially ‘dismissed’ and labeled “junk DNA” [25]. Indeed, soon after the advent of genomic sequencing, reports of mobile elements that were exapted into novel genes and regulatory elements through retrotransposition [26]–[28] or exonization [29] started appearing in the literature. Individual instances of various types of repeat elements were shown to cause disease but to also drive genomic evolution in a positive manner [4],[22]. Recent reports also discuss findings suggesting that the role of mobile elements in genomic evolution, organization and cell process regulation may be significantly more important than previously thought [30]–[34].

Interestingly, the sequences of Alu and B elements are not conserved between human and mouse. For nearly three decades, most searches for regulatory elements made explicit or implicit use of the assumption of equivalence between sequence conservation and function. However, recent work has shown that the human genome regions can be classified into three broad categories with respect to the extent of their evolutionary conservation and their coding potential: (a) sequences that are under *strong evolutionary constraints* (∼5% of the human genome [35],[36]); (b) *conserved non-exonic sequences* that are more frequent than expected [37] but do not necessarily comprise functional elements [38]; and (c) *non-conserved, non-exonic sequences*, a category with an unexpected high number of functional elements [39]. Such findings increasingly question whether sequence conservation is a necessary and sufficient condition for function. Indeed, recent publications have revealed the existence of *regulatory elements* that are not conserved between human and mouse [33], [40]–[45].

Recent studies suggest that RNA silencing pathways including endogenous siRNA and piRNA pathways provide an adaptive defense in the transposon arms race [46], raising the possibility of a connection between RNAi pathway genes and Alu/B element insertions. Key proteins in these pathways, such as Argonaute and PIWI, are categorized as “gene silencing” proteins in the GO hierarchy, a term that is, in fact, identified by our statistical method as significant in the case of antisense upstream B element instances in mouse (see Supplemental Table S4), thus revealing a possible connection among genes that participate in the RNAi pathways and Alu/B elements.

In closing, it is worth emphasizing that, in our analysis, *antisense intronic regions* are significantly more enriched in Alu and B elements than sense intronic regions, unlike upstream regions, where no significant difference is observed between sense and antisense. In view of this finding, and taking into account previous work showing evidence of widespread occurrence of antisense transcription in introns [47],[48] as well as correlation of non-coding antisense intronic RNA levels with tumor differentiation [49], it is reasonable to conjecture that antisense intronic sequences may play an important role in regulation. Conceivably, this conjectured activity may be coordinated with instances of Alu and B elements located upstream of protein-coding genes. Taken together, these findings hint at the existence of a potentially very complex web of interactions among upstream regions, introns, and repeat elements in the context of cell process regulation.

## Materials and Methods

**Data sources.** We obtained genome chromosome sequences and genomic region coordinates for transcripts, exons, introns as well as Gene Ontology (GO) annotations (biological processes and molecular functions) from ENSEMBL release 52. Human/mouse pair-wise alignments and repeat regions corresponding to the same genome assembly versions (NCBI36 for human and NCBIM37 for mouse) were obtained from UCSC Genome Browser.

**Computing densities and associated p-values.** We define density of a given type of elements (for example Alu or B elements) in a given genomic region as the fraction of the region that is covered by the instances of these elements. We calculated the densities of Alu and B elements in genomic regions obtained from all combinations of: (a) distance from gene transcript start positions (1, 2, 4, 8, 16, 32, 64, 128, 256 and 512 kb), (b) direction (upstream and downstream), and (c) orientation (sense and antisense). Each genomic region was identified as follows:

1. for each gene transcript with transcript start position *s*, we identified its upstream (downstream) region at distance *d* as the region covering *d* nucleotides upstream (downstream) of position *s*
2. for each gene transcript, we identified its downstream exonic region at distance *d* as the intersection of its downstream region at distance *d* and its set of exons
3. for each gene transcript, we identified its downstream intronic region at distance *d* as the intersection of its downstream region at distance *d* and its set of introns
4. the final genomic region was determined as the union of all the corresponding gene transcript regions; for example, the upstream region, is the union of the upstream regions of all gene transcripts.

The *expected* Alu and B element densities were calculated on the entire human and mouse genome respectively. All density calculations were performed using *resampling* and the results are shown as mean and standard deviation on Figure 1 for human and Figure 2 for mouse. P-values were computed in each case using Student's T test between the observed and expected, or between sense and antisense in the intronic downstream case. For a wide range of distances (i.e. 4 kb–256 kb), both upstream and downstream, the p-values are practically zero.

**Identifying significant GO terms and computing adjusted p-values.** The following definitions are necessary for the rest of the section. A *genomic locus* x is a quadruplet (x_c_,x_s_,x_a_,x_b_) containing information about its chromosome, strand, and start and stop coordinates. A *genomic region* is a set of genomic loci. The *overlap* θ(x,y) between two genomic loci x and y is θ(x,y) = min(x_b_,y_b_)−max(x_a_,y_a_), if x_c_ = y_c_ and x_s_ = y_s_, and 0 otherwise. The *overlap* θ(Q,R) between two genomic regions Q and R is the sum of overlaps θ(x,y) of all possible pairs (x,y) of genomic loci where x is in Q and y in R. The *density* δ(Q,R) of region Q in reference region R is defined as the overlap θ(Q,R) divided by the total length of reference region R, i.e. the sum of the length of the region's loci.

In order to determine which GO terms are significantly enriched in Alu/B elements, the following information is used as input to our algorithm:

1. the test region Q, i.e. the set of Alu (or B) element genomic loci
2. the reference region r(g) for each gene g, i.e. the set of intronic or upstream genomic loci for each gene
3. the set of genes G(t) associated with each GO term t

For each gene g, we compute the density δ(g) = δ(Q,r(g)) of test region Q in the reference region r(g) of gene g. For each GO term t, we also compute the average density δ(t) of test region Q across the set of reference regions R(x) = { r(g) | g in G(t) }, i.e. the set of reference regions of genes associated with GO term t. Then, we calculate the p-value of δ(t) as the probability p(t) that value δ(t) is drawn from the null distribution. The null distribution of GO term density values is estimated using N = 1,000,000 randomized experiments designed to redistribute the test region loci Q within the reference regions r(g), while satisfying the following criteria:

1. preserve the average test region density across the reference regions, i.e. do not redistribute the test region loci across the entire genome, but instead only inside the reference regions
2. preserve the average test region density in each chromosome and strand
3. preserve the test region loci length distribution in each chromosome and strand
4. most importantly, preserve the variance of test region densities across genes' reference regions

All these criteria can be satisfied by simply permuting the density values δ(g) across genes of the same chromosome and strand. Then, the p-value p(t) for each GO term t is calculated as the number of randomized experiments where the randomized density δ'(t), as computed based on the permuted δ'(g) densities, exceeds or is equal to the observed density value δ(t), divided by the total number of experiments.

Since we carry out only 1,000,000 randomized experiments, p-values smaller than 1e-06 needed to be approximated for presentation purposes in the Supplemental tables, and this was achieved by approximating the tail of the null distribution with an exponential distribution. We point out that all the results presented in the manuscript regarding significance are based on the *exact* p-values and not on the approximated ones. Finally, in order to estimate the false discovery rate (FDR), we computed the adjusted p-values (q-values) according to the method presented in [50]. Two approaches were evaluated: (a) all hypothesis tests were considered as one family, and (b) each level of GO hierarchy was considered as a separate family. The difference of the outcomes of the two approaches was negligible. We also note that for a given repeat element family we analyzed the upstream sense/antisense and intronic sense/antisense regions simultaneously under the same random permutation experiment, i.e. we collected all the gene densities in all four types of regions together, in order to estimate the number of significant GO terms at 1% FDR.

## Supporting Information

Table S1 GO term p-values for human Alu and mouse B elements.
(0.04 MB XLS)

Table S2 GO term p-values and q-values for all human Alu families in upstream regions.
(1.14 MB XLS)

Table S3 GO term p-values and q-values for all human Alu families in intronic regions.
(1.07 MB XLS)

Table S4 GO term p-values and q-values for all mouse B families in upstream regions.
(1.12 MB XLS)

Table S5 GO term p-values and q-values for all mouse B families in intronic regions.
(1.07 MB XLS)

Table S6 GO term p-values and q-values for chimpanzee Alu elements in upstream regions.
(0.50 MB XLS)

Table S7 GO term p-values and q-values for chimpanzee Alu elements in intronic regions.
(0.49 MB XLS)

Table S8 GO term p-values and q-values for rat B elements in upstream regions.
(0.54 MB XLS)

Table S9 GO term p-values and q-values for mouse B elements in intronic regions.
(0.53 MB XLS)
